# Towards automated fetal brain biometry reporting for 3-dimensional T2-weighted 0.55-3T magnetic resonance imaging at 20-40 weeks gestational age range

**DOI:** 10.1007/s00247-025-06403-2

**Published:** 2025-11-14

**Authors:** Aysha Luis, Alena Uus, Jacqueline Matthew, Sophie Arulkumaran, Alexia Egloff Collado, Vanessa Kyriakopoulou, Sara Neves Silva, Jordina Aviles Verdera, Megan Hall, Simi Bansal, Sarah McElroy, Kathleen Colford, Jo Hajnal, Jana Hutter, Lisa Story, Mary Rutherford

**Affiliations:** 1 Department of Early Life Imaging, School of Biomedical Engineering & Imaging Sciences, King’s College London, St Thomas’ Hospital, London, SE1 7EH UK; 2 Barts Health NHS Trust, London, United Kingdom; 3 St George’s University Hospitals NHS Foundation Trust, London, United Kingdom; 4 Guy’s and St Thomas’ NHS Foundation Trust, London, United Kingdom; 5 Siemens (United Kingdom), Camberley, United Kingdom; 6 Universitätsklinikum Erlangen, Erlangen, Germany

**Keywords:** Fetus, Neuroimaging, Biometry, Magnetic resonance imaging, Deep learning

## Abstract

**Background:**

The detailed assessment of fetal brain maturation and development involves morphological evaluation, gyration analysis, and reliable biometric measurements. Manual measurements on conventional 2-D magnetic resonance imaging (MRI) are affected by fetal motion, and there is no clear consensus regarding definitions for brain biometric parameters and anatomical landmark placements, making consistent reference plane and slice selection challenging. Automated biometry with 3-D slice-to-volume reconstruction (SVR) has the potential to improve the reliability of derived measurements, allowing precise quantification of fetal brain development. Previous published works have primarily focused on the technical feasibility of automated fetal brain biometry methods for T2-weighted (T2W) MRI. However, none have proposed solutions for automating the reporting of biometry results, which could enhance clinical utility and support real-time integration into routine clinical workflows. Furthermore, there is no consensus on a universal fetal biometry protocol for 3-D fetal MRI.

**Objective:**

To implement and validate a fully automated biometry reporting pipeline for 3-D T2W fetal brain MRI, based on deep learning biometry measurements and computation of *z*-scores and centiles, by comparison to normative growth charts.

**Materials and methods:**

Automated extraction of 13 routinely reported linear fetal biometry measurements using deep learning localization of anatomical landmarks in 3-D reconstructed T2W brain images based on 3-D UNet and presentation of the results in an .html report with centile calculation. The automated biometry method was quantitatively evaluated on 90 retrospective cases against expert manual measurements. Additionally, the fully automated, end-to-end biometry reporting pipeline was prospectively evaluated on 111 cases across a wide range of gestational ages, field strengths, and scanning parameters. We also generated normal centile ranges for 19-40 weeks GA range from 406 normal control datasets.

**Results:**

The retrospective quantitative evaluation demonstrated good agreement with manual measurements, with the maximum absolute difference between automated vs. manual measurement within a 1-3-mm range. In the prospective evaluation, more than 98% of landmark placements were graded as acceptable for interpretation and measurements. The processing time of the pipeline was less than 5 min per case, with measurements and centiles available at the time of reporting. Inspection of the automated landmark placement and computed biometrics took 1-3 min per case. The generated normative growth charts demonstrate strong correlation with the trends in the previously reported works.

**Conclusion:**

Our approach is the first to develop a fully automated biometry reporting pipeline for 3-D T2-weighted fetal MRI which integrates deep learning-based measurements, centile and *z*-score calculation vs. normative growth charts and report generation.

**Graphical abstract:**

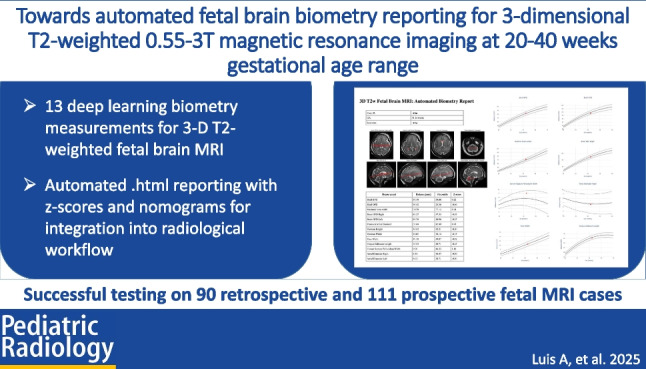

## Introduction

The role of fetal magnetic resonance imaging (MRI) has grown exponentially in recent years, and is currently used for detailed assessment, prognostication, and treatment planning [1, 2] across an increasing range of indications, including fetal brain development [3, 4], fetal body anomalies [5, 6], congenital heart disease[7], placental pathologies [8] and high-risk pregnancies.

Accurate and detailed assessment of fetal brain development involves morphological evaluation, gyration analysis, and reliable biometric measurements. This enables early detection and characterization of several central nervous system (CNS) anomalies, allowing comprehensive antenatal and postnatal evaluation. It also provides a clearer understanding of the severity and impact of detected abnormalities, leading to increased parental choices and improved neonatal and childhood outcomes [9].

In current clinical practice, biometric MRI measurements are usually performed manually on 2-dimensional (2-D) slices, based on widely available methodology and reference biometric charts [10, 11]. There are, however, inherent challenges of acquiring accurate measurements on 2-D slices. Fetal and maternal motion can result in suboptimal acquisition planes, as well as inter-slice and inter-plane motion, which can lead to false-positive or false-negative results and contribute to the uncertainty in the obtained measurements. These technical challenges are particularly pronounced at early gestational ages (GA), where increased fetal movement and small brain structures make them especially susceptible to measurement error. Although additional reference biometric charts have since been published for the posterior fossa [12, 13], cavum septum pellucidum [14], and for early GA (20-24 weeks) [15], they also rely on measurements from 2-D slices.

Three-dimensional (3-D) slice-to-volume (SVR) reconstruction [16, 17] of the fetal brain addresses many of the technical challenges by enabling full volumetric assessment, better visualization of anatomy, and reliable biometric measurements that are concordant with those obtained from true orthogonal 2-D acquisition planes [18–20].

Currently, there is significant variation in practice across centers, with no clear consensus on several aspects of fetal brain biometry. These include the definition of anatomical landmarks for each measurement, selection of the reference planes and slices, determining which structures and parameters should be measured to assess normal growth and diagnose specific pathologies, and deciding whether inner or outer anatomical boundaries should be used. The impact of partial volume effects and asymmetry of brain structures also remains inadequately addressed. Furthermore, there is a well-known and expected degree of variance in manual measurements, further limiting the consistency and accuracy of biometry assessments.

The lack of universally accepted biometry measurement definitions and methodology across different GAs and field strengths highlights the need for detailed, systematic, and standardized guidelines that can serve as a baseline. Indeed, a systematic review focused on posterior fossa measurements in fetal MRI [21] identified 62 distinct 2-D biometric measurements, many of which assessed similar features using slightly different methods. Another systematic review [22] highlighted that the existing MRI reference ranges for fetal brain biometry have low-to-moderate methodological quality, emphasizing the need for a more robust, standardized approach. Similar findings were reported in a further systematic review, demonstrating heterogeneity in methods of existing fetal corpus callosum biometric reference charts [23].

Automated biometry has the potential to improve the reliability and reproducibility of measurements, allowing precise quantification of fetal brain development. A few recent studies have demonstrated the technical feasibility of automating biometry for various fetal MRI measurements. The works of Avisdris et al. [24] and She et al. [25] demonstrated automated biometry in 2-D coronal slices using a combination of deep learning brain segmentation and machine learning morphological operations for plane selection and extraction of three linear brain measurements. Matthew et al. [26] applied atlas-based registration for propagation of landmarks to extract 31 craniofacial biometry measurements from 3-D SVR fetal MRI head images. Masterl et al. [27] proposed deep learning for the detection of landmarks and extraction of 11 brain measurements in 3-D SVR brain images.

However, to our knowledge, no reporting pipeline currently exists which could automate 3-D SVR T2W MRI derived biometry measurements, centile calculations, and normative growth charts, and generate a clinically relevant and actionable report. Implementing reliable automated biometry directly during MRI examinations could significantly enhance clinical utility and streamline workflows, reducing the time spent acquiring manual measurements. This capability is essential for real-time integration into routine clinical practice.

## Contributions

This work introduces the first prototype solution for automated reporting of brain biometry for 3-D T2w fetal MRI based on deep learning extraction of measurements and calculation of *z*-scores by comparison to normative growth models. The outputs are presented in a structured.html report designed for integration into the radiological workflow and mirrors the standardized formats routinely used in antenatal ultrasound software. The added value is automation of conventional manual biometric measurements which are time consuming and affected by intra- and inter-observer variability. This will potentially contribute to improved standardization and workflow efficiency.

The main development steps include formalization of biometry protocol based on current clinical practice at our institution, implementation of deep learning-based landmark pipeline for computation of 13 standard biometric measurements, and automated reporting of centiles using nomograms generated from 406 control datasets across 0.55-3-T field strength and 19-40 GA. The performance of the deep learning biometry method is quantitatively evaluated on 90 retrospective datasets by comparison with manual measurements. In addition, the clinical utility of the full reporting pipeline is qualitatively evaluated on 111 prospective datasets through quality scoring of landmark placement.

## Materials and methods

### Cohort, and image acquisition and preprocessing

- A total of 203 fetal datasets acquired on 3-T Philips Achieva MRI system (Philips Healthcare, Best, The Netherlands) using a 32-channel cardiac coil with echo time=180 ms, acquisition resolution 1.25 × 1.25 mm, slice thickness 2.5, −1.5-mm gap, and 5-6 stacks;
- A total of 39 fetal datasets acquired on 1.5-T Philips Ingenia MRI system (Philips Healthcare, Best, The Netherlands) using a 28-channel torso coil with echo time=180 ms, acquisition resolution 1.25 × 1.25 mm, slice thickness 2.5, −1.25-mm gap, and 4-5 stacks;
- A total of 72 fetal datasets acquired on 1.5-T Siemens Sola MRI system (Siemens Healthineers, Erlangen, Germany) using two 30- and 18-channel body coils with echo time=80 and 180 ms, acquisition resolution 1.24 × 1.25 mm, slice thickness 3 mm, and 9-12 stacks;
- A total of 212 fetal datasets acquired on 0.55-T Siemens MAGNETOM Free.Max MRI system (Siemens Healthineers, Erlangen, Germany) using a six-element flexible coil and a nine-element spine with echo-time=105–106 ms, acquisition resolution 1.48 × 1.48 mm, slice thickness 4.5, and 9-12 stacks [28].

The inclusion criteria for the training and testing cohorts were the following:

1. Healthy volunteer,
2. Singleton pregnancy,
3. More than four fetal MRI stacks with full brain coverage,
4. Acceptable SVR reconstruction quality [17] with sufficient visibility of the major brain structures and tissue interfaces, and
5. Normal brain appearances. Additional criteria for the prospective cohort, which included a subset of cases referred for clinical indications, was the absence of reported anomalies.

The cohorts included a total of 525 MRI examinations from 18-41 weeks GA, including 414 retrospective and 111 prospective datasets. Training of the deep learning model was conducted on 150 datasets followed by testing on 90 retrospective datasets. We used 406 retrospective normal control datasets for generation of normative growth charts (which included most of the training and testing cases). The prospective evaluation of the pipeline was performed on 111 additional prospectively acquired datasets that were not used in any other pipeline development steps. All cases were de-identified prior to processing through the pipeline.

**Fig. 1:**
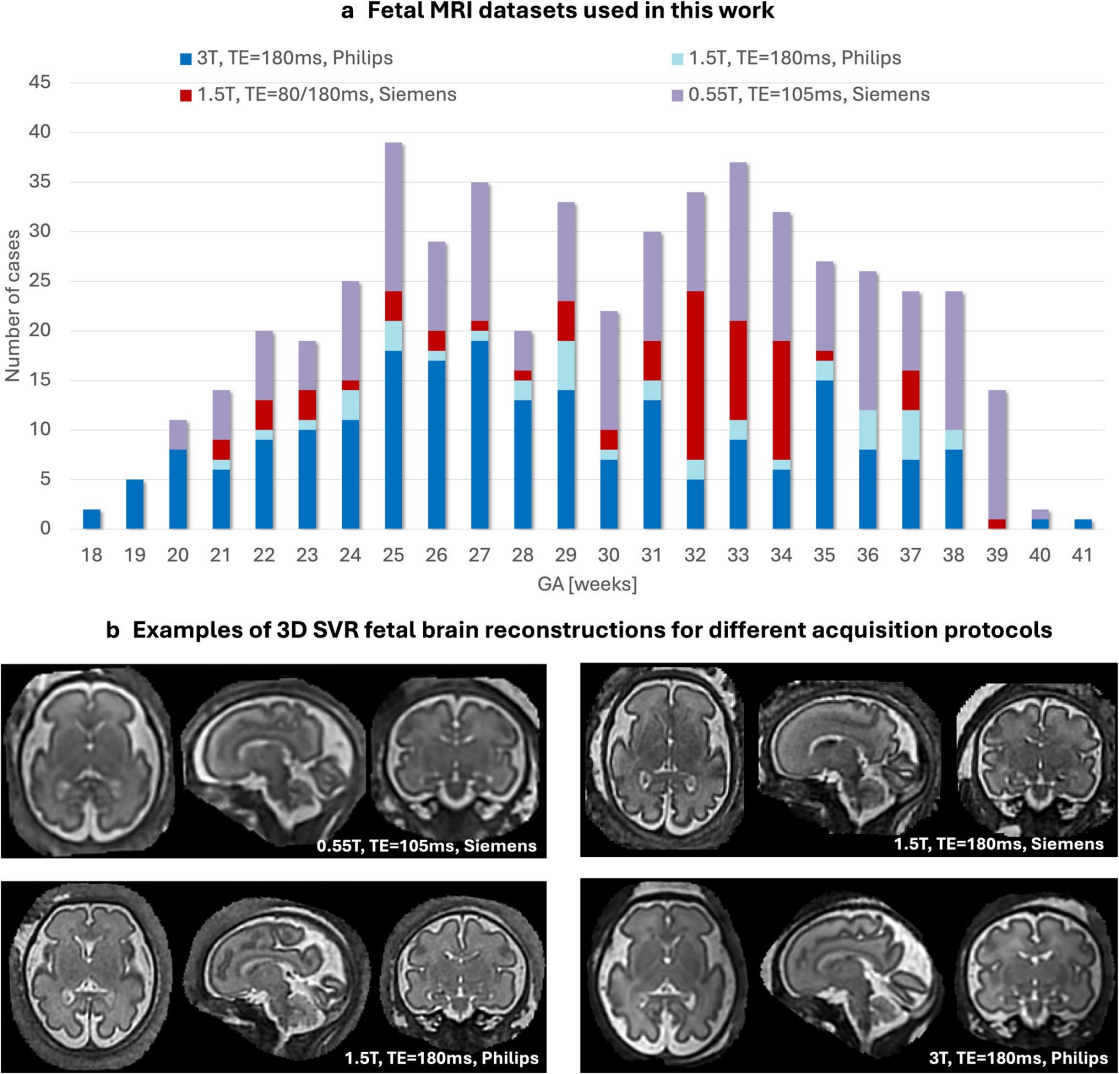
**a** GA distribution of 525 fetuses imaged at 0.55 T, 1.5 T, and 3 T with T2W datasets used for training of the deep learning model, generation of growth charts, retrospective quantitative testing, and prospective qualitative evaluation. **b** Examples of 3-D SVR fetal brain reconstruction for different acquisition protocols. *3-D* 3-dimensional, *GA* gestational age, *MRI* magnetic resonance imaging, *SVR* slice-to-volume registration, *T* Tesla, *TE* echo time, *T2W* T2-weighted

The majority of the datasets used in this work (Fig. 1) were acquired on 3 T (40*.*4%) and 0.55 T (38*.*7%). The smaller proportion of the 1.5-T Ingenia Philips datasets (7*.*4%) was due to lower availability of normal control cases acquired on this scanner. The 1.5-T Sola Siemens datasets were used only in the prospective evaluation, and the proportion of cases (13*.*5%) corresponds to the number of research-consented clinical cases without brain anomaly findings.

All images were 3-D reconstructed using the fully automated auto-proc-SVRTK^1^ pipeline [17, 29, 30] to 0.8-1.0-mm resolution in the standard radiological space. In addition, 3-D brain tissue segmentation was performed on each image volume using the “brain volumetry and automated parcellation for 3-D fetal MRI” (BOUNTI) method [31].

### Formalization of landmark-based biometry protocol

The current clinical practice at our institution involves 2-D biometric measurements taken on 3-D SVR images, as this approach provides full brain coverage and volumetric assessment, which is essential for reliable biometry [19].

We aimed to expand on the framework defined by Kyriakopoulou et al. [19] through workshops with experienced reporting radiologists specializing in fetal MRI (MR, AE, SA, AL) with the goal of establishing a reproducible biometric protocol. For each biometric parameter, the anatomical landmarks, reference planes, and slices were clearly defined across all GAs, accounting for partial volume effects.

Each measurement was then defined during the consensus meetings on a subset of cases at 20-35 weeks GA using a landmark-based approach with 3-D-sphere point labels manually created in ITK-SNAP.^2^

We incorporated additional measurements including the anteroposterior diameter of the pons, corpus callosum length, and cavum septum pellucidum width. Skull measurements were also updated to reflect outer-to-outer boundaries [32, 33]. These additional parameters were selected due to their relevance in monitoring normal fetal growth and development [34].

Our finalized protocol includes 13 routine linear measurements of the skull, brain, ventricles, corpus callosum, cerebellum, pons, and vermis (including left and right paired measurements to account for potential asymmetries).

### Automated brain biometry pipeline

**Fig. 2:**
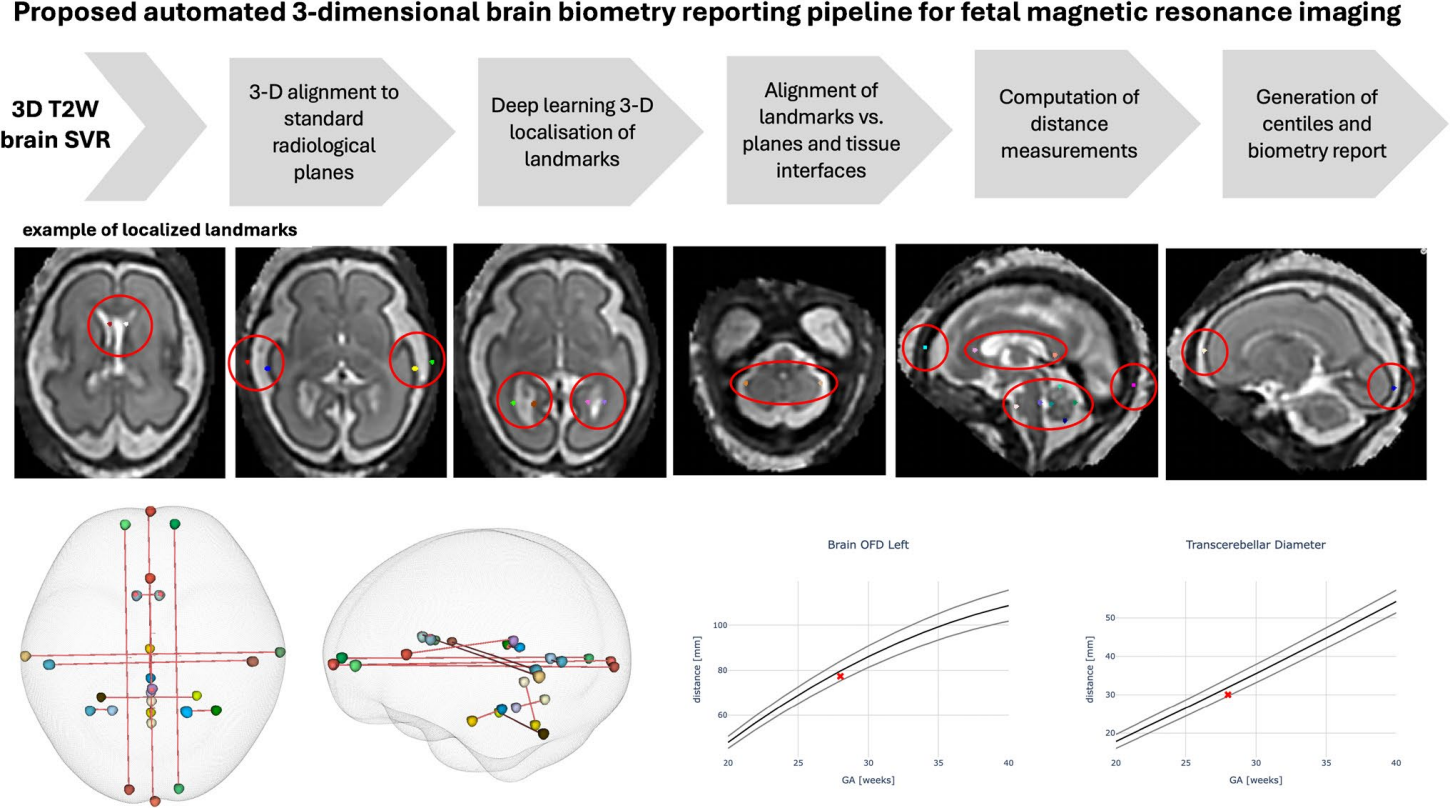
Proposed pipeline for automated brain biometry for 3-D T2W fetal MRI. *3-D* 3-dimensional, *OFD* occipito-frontal diameter, *SVR* slice-to-volume reconstruction, *T2W* T2-weighted

The proposed pipeline for automated biometry for 3-D T2W fetal brain images is illustrated in Fig. 2. First, 3-D SVR-reconstructed T2W brain images are aligned to the standard radiological planes and resampled to 0.5-mm resolution. This is followed by deep learning localization of biometric landmarks via segmentation of the corresponding 26 3-D sphere labels. For segmentation, we use a classical 3-D UNet architecture [35] implemented in the MONAI [36] framework. The preprocessing includes masking of the brain and affine atlas registration [37] performed using MIRTK toolbox^3^. The network was trained on 150 datasets (0.55-T and 3-T) covering 18-41 weeks GA. The training landmark labels were generated through manual refinement of preliminary network outputs in ITK-SNAP. All segmented landmarks were postprocessed by additional alignment to the relevant planes and tissue interfaces of brain tissue labels. The final biometry measurements were computed in millimeters as distances between 3-D landmark coordinates. All steps are combined into a single script, which is publicly available in the *auto-proc-SVRTK* repository https://github.com/SVRTK/auto-proc-svrtk.

### Normative growth charts for fetal brain biometry

In order to provide reference ranges for the reporting tool, we used the proposed pipeline to generate normative biometry growth chart models with 5th, 50th, and 95th centiles from 406 healthy control datasets (including 0.55 T, 1.5 T, and 3 T) from 18 to 40 weeks GA range. All automated landmarks were reviewed and manually refined, if required. The models were generated using classical quadratic polynomial fitting [38].

### Automated biometry reporting and normative growth charts

The integration of the proposed automated biometry pipeline into routine clinical practice would require an intuitive output format, such as a report, which is easy to interpret and supports further analysis on an individual case basis, thereby enhancing the real-world applicability and acceptability.

Therefore, after the landmark detection step, the re-oriented 3-D brain image and 3-D biometry landmark files were passed to the python script that automatically generated an.html report page. The report displays:

- General information about the case (patient number, scan date, and GA);
- Eight representative 3-D brain SVR images with automated landmark-based 2-D linear measurements overlaid;
- Table with extracted biometry measurements, computed centiles, and *z*-scores;
- Growth charts of 13 extracted measurements vs. normative curves with centiles;
- Disclaimer that the current report is for research purposes only and hence should not be used as a diagnostic tool (awaiting regulatory approval for routine clinical use).

The full pipeline combining automated biometry and reporting scripts is publicly available as a standalone application via auto-SVRTK^4^ docker operational on Windows, MAC, and Linux systems. The source code with instructions is publicly available online at the auto-SVRTK^5^ GitHub repository.

### Evaluation

The general feasibility of the implemented automated biometry pipeline was first quantitatively tested on 90 retrospective cases using control datasets acquired with three protocols at 0.55-T, 1.5-T, and 3-T field strengths and covering a 22-38 week GA range. For all cases, two sets of manual linear measurements were performed by experienced neuroradiologists (MR, AE, SA, AL) and a researcher in fetal MRI (AU). Comparisons were performed between manual measurements, and between automated measurements and average manual measurements. The differences were computed as absolute errors (in mm).

Next, we conducted qualitative prospective evaluation of the clinical utility and acceptability of the pipeline on 111 prospective cases acquired at St Thomas’ Hospital London between July and December 2024. These datasets were not used in training, retrospective performance testing or generation of the normative growth charts. Inclusion criteria included consent for research participation (healthy volunteers and clinical referrals), singleton pregnancy, 20-38 weeks GA range, good reconstruction quality with clear visibility of brain structures, and absence of extreme structural abnormalities. For each case, the usability of the automated biometry report was assessed, and all automated landmark placements were visually rated on a Likert scale by four experienced neuroradiologists (MR, AE, SA, AL; 4-30 years experience in fetal and neonatal MRI) and one researcher in fetal MRI (AU; 6 years of experience). Additionally, end-user feedback was collected to evaluate perceptions regarding the pipeline’s efficiency, impact on workload, and overall confidence levels.

Manual measurements and review of landmark placements were performed in ITK-SNAP, 3-D Slicer^6^, and MITK Workbench^7^ platforms for medical image visualization.

## Results

### Landmark-based fetal brain biometry protocol

**Fig. 3:**
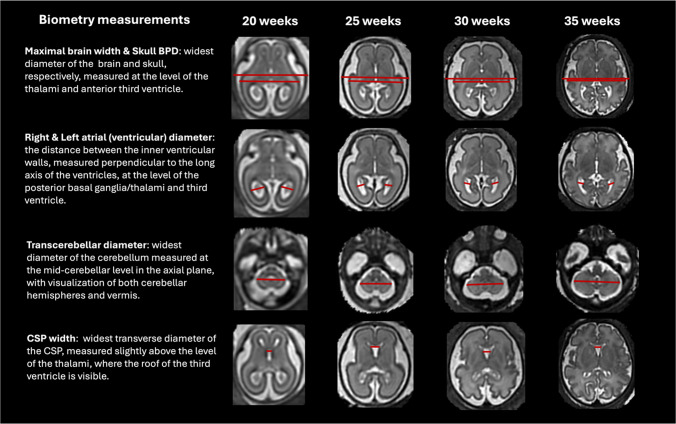
Proposed formalized protocol for automated brain biometry for 3-D T2W fetal MRI from 20 to 35 weeks GA range (axial planes, part 1). *BPD* bi-parietal diameter, *CSP* cavum septum pellucidum

**Fig. 4:**
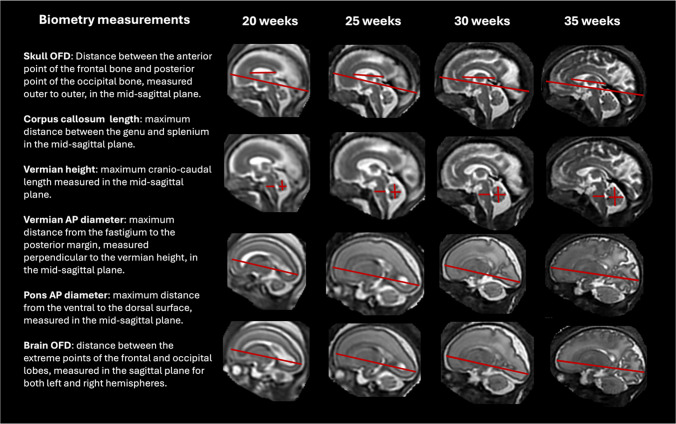
Proposed formalized protocol for automated brain biometry for 3-D T2W fetal MRI from 20 to 35 weeks GA range (sagittal planes, part 2). *AP* antero-posterior, *OFD* occipito-frontal diameter

Figures 3 and 4 present the proposed standardized protocol with definitions of 13 routine fetal measurements (including left and right atrial diameters and brain occipito-frontal diameter) and representative images at 20-35 week time points.

The biometric parameters were chosen for their relevance in monitoring normal fetal growth and development, supporting the diagnosis of common abnormalities such as ventriculomegaly, corpus callosum agenesis, and posterior fossa malformations, as well as prognosis prediction. It builds on the work outlined by Kyriakopoulou et al. [19], updating measurement definitions to reflect the current standard clinical practice at our institution, including renaming brain biparietal diameter to maximal brain width. Additionally, key parameters routinely measured were incorporated. The updated definitions also account for the dynamic morphological changes with gestation, partial volume effects, and variations in field strength.

- The skull biparietal diameter (BPD) is defined as the widest diameter of the skull and is measured in the axial plane at the level of the thalami and anterior third ventricle, from outer to outer.
- The skull occipito-frontal diameter (OFD) is defined as the distance between the anterior point of the frontal bone and the posterior point of the occipital bone, measured in the mid-sagittal plane from outer to outer.
- The maximal brain width is the widest diameter of the brain, measured at the level of the thalami and anterior third ventricle in the axial plane.
- The brain OFD is the distance between the extreme points of the frontal and occipital lobes, measured separately for the left and right hemispheres in the sagittal plane.
- The right and left atrial (ventricular) diameters are measured as the distance between the inner ventricular walls, perpendicular to the long axis of the ventricles, at the level of the posterior basal ganglia, thalami, and third ventricle and posterior to the choroid plexus.
- The cavum septum pellucidum (CSP) width is defined as the widest transverse diameter of the CSP, between the inner margins of the septal leaflets. It is measured slightly above the level of the thalami, where the roof of the third ventricle is visible.
- The transcerebellar diameter (TCD) is defined as the widest diameter of the cerebellum and is measured at the mid-cerebellar level in the axial plane, ensuring visualization of both cerebellar hemispheres and the vermis.
- The vermian height is the maximum cranio-caudal length, and the vermian antero-posterior (AP) diameter is the maximum distance from the fastigium to the posterior margin, measured perpendicular to the vermian height, both obtained in the mid-sagittal plane.
- The pons antero-posterior diameter is the maximum distance from the ventral to the dorsal surface, measured in the mid-sagittal plane.
- The corpus callosum length is the maximum distance between the genu and splenium, measured in the mid-sagittal plane.

### Retrospective evaluation of the automated biometry pipeline

**Fig. 5:**
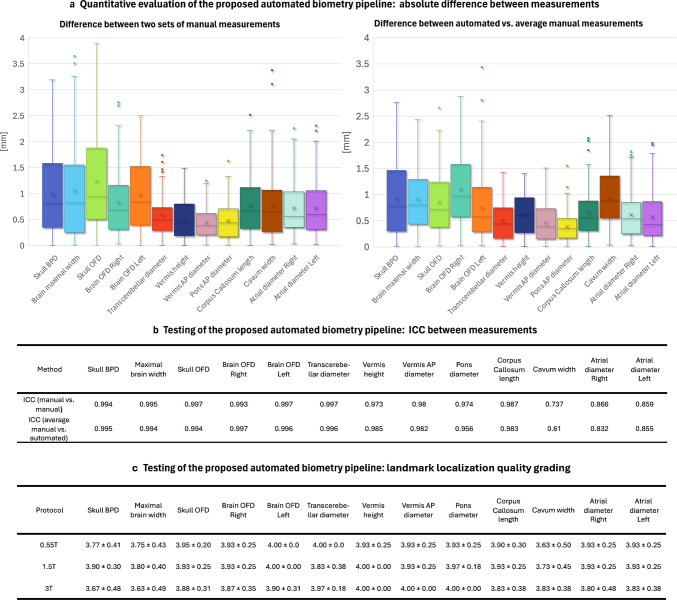
Testing of the proposed automated biometry pipeline on 90 0.55-T/1.5-T/3-T cases from 22 to 38 weeks GA. **a** Absolute difference between two sets of manual measurements and absolute difference between average manual vs. automated measurements. **b** ICC values between two sets of manual measurements and between average manual vs. automated measurements. **c** Landmark localization quality grading (*4* good; *3* acceptable; *2* poor; *1* fail). *AP* antero-posterior, *BPD* bi-parietal diameter, *GA* gestational age,* ICC* intra-class correlation coefficient, *OFD* occipito-frontal diameter, *T* tesla

**Fig. 6:**
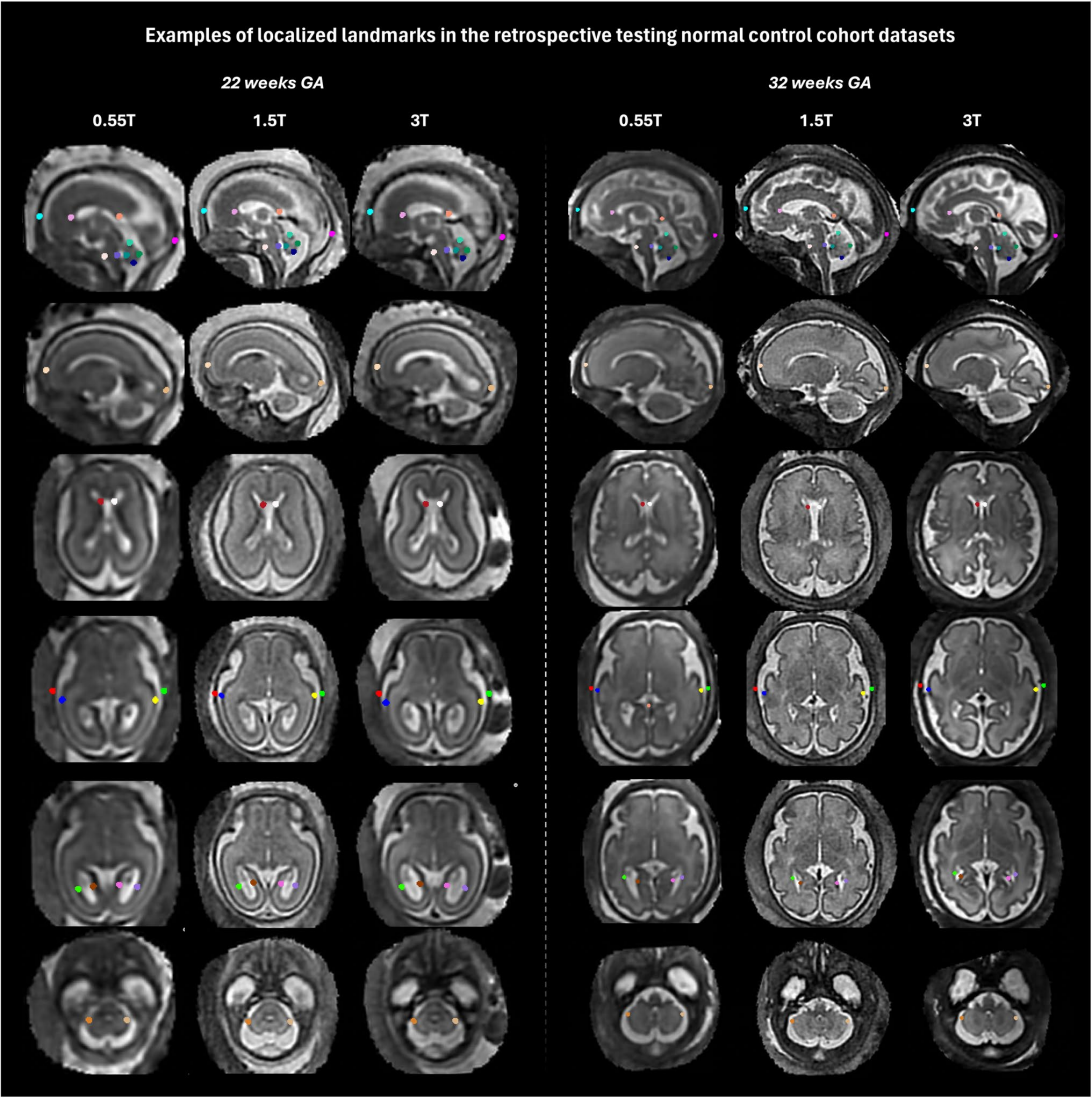
Retrospective evaluation of the proposed automated biometry pipeline on 90 0.55-T/1.5-T/3-T cases from 22 to 38 weeks GA: examples of localized landmarks in different acquisition protocols and GAs. *GA* gestational age, *T* tesla

Figure 5 summarizes the results of retrospective quantitative evaluation of the pipeline on 90 cases acquired with 0.55-T, 1.5-T, and 3-T protocols and covering 22-38 weeks GA range for automated vs. two sets of manual measurements. The intra-class correlation coefficient (ICC) values demonstrated good to excellent reliability for most measurements, and are comparable between both manual vs. manual and automated vs. average manual measurements for all 13 biometrics. The distribution of absolute errors was also similar between manual measurements with the presence of several outliers. The maximum absolute errors compared to manual measurements ranged from 1 to 3 mm, consistent with previously reported variability in automated methods [24, 25, 27]. Examples of localized landmarks for test cases at different acquisition protocols and GAs are shown in Fig. 6. The placement of automated landmarks was rated (AL) as good or acceptable in all cases. The measurements with the highest degree of uncertainty were the maximal brain width, ventricular (atrial) diameters, and cavum width due to the anatomical variability and the absence of fixed structural landmark points in 3-D, even after protocol formalization. Atrial diameters and cavum width also showed lower ICC for inter-observer variability. This indicates that while these results confirm general feasibility of the automated biometry vs. manual measurements, further work is required to establish a biometry protocol specifically adapted for 3-D SVR images. Moreover, further model optimization for anatomical variations along with incorporation of automated quality control for image quality and landmark placement certainty grading will be required to achieve stable performance.

### Normative growth charts for fetal brain biometry

**Fig. 7:**
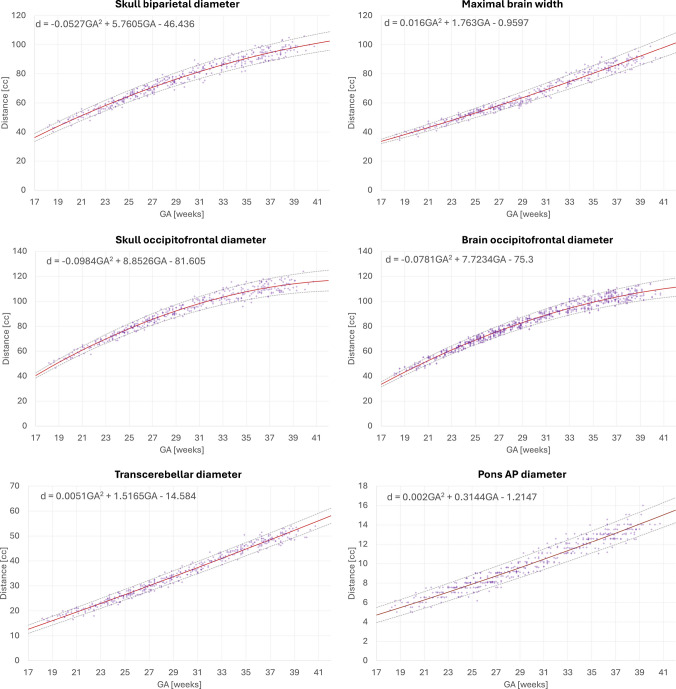
Normative growth charts created from automated biometry for 406 control (0.55 T, 1.5 T, and 3 T) subjects with centiles (part 2). *AP* antero-posterior, *GA* gestational age

**Fig. 8:**
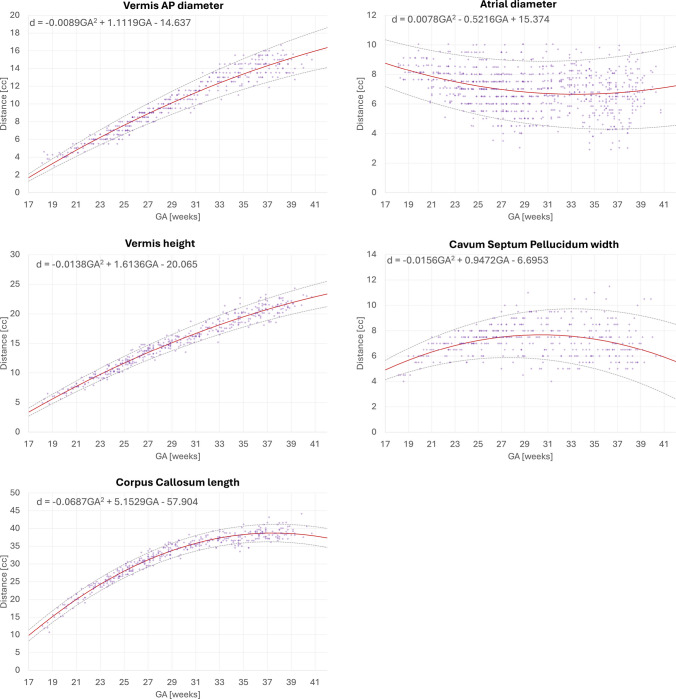
Normative growth charts created from automated biometry for 406 control (0.55 T, 1.5 T, and 3 T) subjects with centiles (part 1). *AP* antero-posterior, *GA* gestational age

Figures 7 and 8 display the growth charts and quadratic normative models generated from automated biometry measurements of 406 normal control datasets spanning 18 to 40 weeks GA range. All biometry results were inspected and minor manual corrections of the landmarks were required in 53 cases due to suboptimal image quality or low visibility, which did not result in significant difference on the trendlines. The numerical scales and shape characteristics of the models correlated strongly with the trends previously reported by Kyriakopoulou et al. [19] and have a similar appearance to the trends in earlier guidelines [10–13, 15]. All global measurements, including skull BPD, skull OFD, brain OFD, and brain maximal width, increase with GA with growth trajectories slightly decelerating towards late gestation. The transcerebellar diameter, pons width, vermian AP diameter, and vermian height trends show nearly linear growth. The corpus callosum length tends to plateau after approximately 33 weeks. The ventricle (atrial) diameters demonstrated high variance across the entire GA range, consistent with previously works  [19]; the global trend demonstrates that ventricles are larger at earlier GA with a tendency to stabilize at later gestation. There is also a high variance in cavum width which plateaus after the third trimester.

The documents with centile model formulas based on the mixed acquisition protocol datasets together with examples of the landmark biometry protocol defined in the atlas space are publicly available online in a dedicated repository https://gin.g-node.org/ kcl cdb/fetal mri biometry.

### Prospective evaluation of the biometry reporting pipeline

The prospective qualitative evaluation was successfully performed on 111 prospectively acquired datasets without reported extreme structural brain anomalies. Qualitative grades and comments were collected from evaluators regarding landmark placement (26 in total) and general end-user feedback on the utility and practicality of the reporting pipeline.

**Fig. 9:**
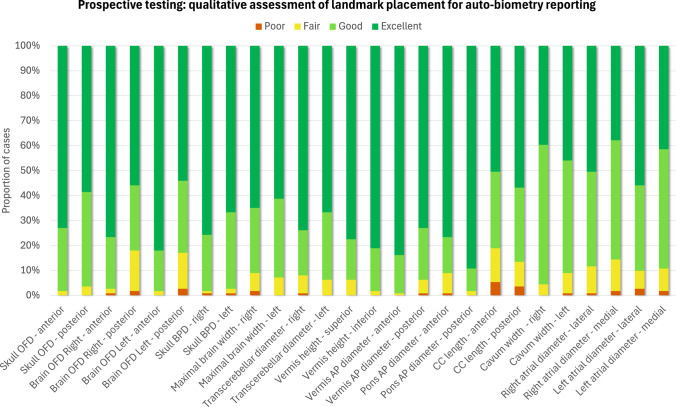
Results of qualitative prospective evaluation of landmark placement for 111 prospectively acquired datasets from 0.55-T, 1.5-T, and 3-T acquisition protocols. Quality grades: poor, fair, good, excellent. *AP* antero-posterior, *BPD* bi-parietal diameter, *CC* corpus callosum, *OFD* occipito-frontal diameter

The resulting average quality scores for each of the 26 landmarks across 13 measurements are presented in Fig. 9. Overall, more than 98% of landmark placements were graded as acceptable for interpretation and measurements (64*.*2% “excellent”; 28*.*2% “good”; 6*.*5% “fair” grades). Only 1.1% of landmarks were graded as “poor.” The highest proportion of “excellent” grades were observed for skull OFD and BPD, TCD, pons and vermis AP diameters, and vermis height. This can be attributed to the pronounced and clearly visible anatomical features that are required for accurate placement of landmarks. In comparison, the maximal brain width and brain OFD landmarks received a higher proportion of “good” grades, as expected, due to greater morphological variation and increased cortical folding that occur with gestation. The lowest ratings, but still graded as “good,” were observed for cavum and atrial diameter measurements due to anatomical variability, and corpus callosum length due to suboptimal visibility of the genu.

These results reaffirm that using automated biometry reporting in clinical practice is feasible and suggest that the proposed pipeline could potentially serve as a baseline for further adaptation and retraining of the model across a wide range of brain anomalies. However, the minor proportion of low-quality grades highlights that automated measurements should always be visually inspected and corrected, when required, prior to reporting.

**Fig. 10:**
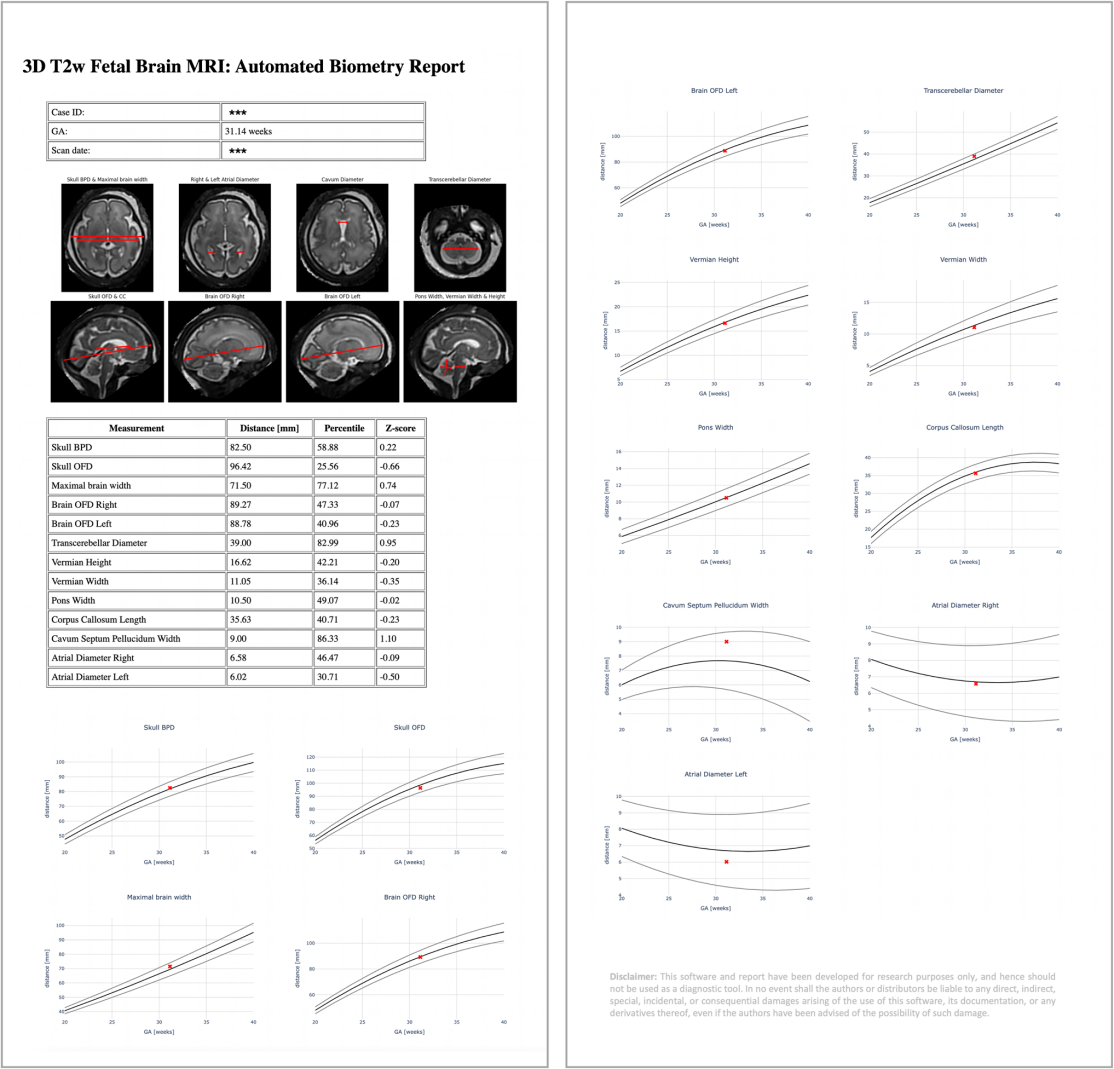
Example of the automated report for a test case generated as an .html file. *3-D* 3-dimensional, *BPD* bi-parietal diameter, *CC* corpus callosum, *GA* gestational age, *ID* identification, *MRI* magnetic resonance imaging, *OFD* occipito-frontal diameter, *T2W* T2-weighted

An example of the generated automated biometry.html report is shown in Fig. 10. The format was confirmed as acceptable for clinical interpretation. The total processing time for the full pipeline including preprocessing, deep learning biometry, and report generation is less than 5 min per case on average which is within reasonable ranges for reporting.

## Discussion

The main aim of this work was the automation of conventional biometric measurements and *z*-score calculations for fetal brain MRI, which are currently performed manually during radiological reporting. It introduces the first prototype of a fully automated, end-to-end pipeline for brain biometry and centile calculation in 3-D motion-corrected T2W fetal MRI across different field strengths and GA ranges, within a clinical reporting pipeline.

We first established the landmark-based protocol with 13 routine linear measurements (including paired measurements) through expert consensus with experienced fetal neuroradiologists. Each measurement was clearly defined by determining precise and reproducible anatomical landmarks on reference planes and slices, and considering GA-related changes in morphology of the structures being measured. Next, we developed a deep learning pipeline to segment the selected landmarks for each biometric parameter and automate the measurements, which was trained on 150 cases from different acquisition protocols and a wide GA range. We implemented a script for automated reporting that displays the biometry results and centile calculation, with the output presented in an .html format, available to the end-user at the time of reporting as a decision support tool to help guide accurate and consistent biometry assessment. The performance of the pipeline was quantitatively evaluated on a retrospective test cohort of 90 cases, and its clinical utility and acceptability were qualitatively assessed on a prospective cohort of 111 cases.

Our results demonstrate good accuracy, with the range of absolute errors compared to manual measurements varying from 1-3 mm, consistent with the previously reported variability in automated fetal brain biometry methods. The prospective evaluation assessing the placement of each landmark was also considered good or excellent for more than 90% of all landmarks in all datasets. In both retrospective testing and prospective evaluation, the measurements with the highest degree of variability were the maximal brain width, corpus callosum length, ventricular atrial diameters, and cavum width. While we defined the landmarks across all measurements, the absence of fixed and clearly visible anatomical structures for the above parameters may have introduced inconsistencies in model identification and subsequently reduced reproducibility. Other factors contributing to variability include partial volume effects, image quality, and normal anatomical variability. However, for the smaller brain structures, such as the vermis and pons, the results were favorable, with consistent quality assessments rated as good or excellent. These structures have traditionally posed challenges for accurate assessments on 2-D slices due to the difficulty in obtaining true orthogonal mid-sagittal planes. The ability to reliably assess such measurements demonstrates the potential for improved biometry accuracy using automation with SVR reconstructions. Furthermore, the average time to complete all manual measurements is 10-15 min for an experienced fetal neuroradiologist, while inspection of the automated landmark placement and computed biometrics took only 1-3 min per case. Therefore, even in cases where certain parameters may require re-measurement, the overall time savings and efficiency gains are substantial.

The main strengths of this work include a large normative dataset which spans a wide GA range, encompasses diverse acquisition parameters, and incorporates 3-D SVR. The utilization of 3-D SVR reconstruction has shown to improve the accuracy of fetal brain biometry by addressing the technical limitations of traditional 2-D slice based measurements [18, 19].

The original methodology for biometry measurements for fetal brain MRI [10] was proposed more than 20 years ago on 2-D slices, developed in accordance with ultrasound practices. It was also derived from fetuses referred for suspected abnormalities, on single field strength with limited sample sizes at extreme GAs. Accurate assessments on 2-D images rely on the acquisition of true orthogonal planes, which are often challenging due to motion. Furthermore, the optimal slice may not always be captured as a result of inter-slice motion, resulting in under- or overestimation of the measurement.

A further limitation of this methodology is that it does not address the dynamic changes in fetal brain morphology, making it difficult to apply consistent reference points across different GA. For example, the work by Garel [10] defines the measurement of the lateral ventricles as being taken at the level of the atria (with good visibility of the choroid plexuses), on an axis perpendicular to that of the ventricle and at the mid-height of the ventricle. However, at younger GAs, the orientation of the ventricles is more vertical making it difficult to use the same axis defined for later gestation when the shape adopts the adult-like orientation.

By refining and expanding on the biometry protocol proposed by Kyriakopoulou et al. [19], our work addressed this challenge by accounting for the developmental changes in brain structures, demonstrating reproducible automated biometry across different GAs.

Moreover, given the vast amount of structural information that can be extracted from fetal MRI scans, establishing MRI-specific normative reference ranges would not only improve the accuracy and reliability of biometric measurements, but also capture the complex and dynamic process of fetal brain development. The generated normative growth charts based on the proposed automated biometry pipeline from 406 control subjects across 18-40 weeks GA range demonstrate high correlation with the trends in the previous works.

Compared to previous published work on automated biometry, which narrowly focus on technical performance, we have developed an enhanced solution by implementing a comprehensive end-to-end pipeline. We have also demonstrated the feasibility of this pipeline in a prospective clinical setting, facilitating real-time integration and streamlining clinical workflows, offering operational benefits such as improved efficiency.

Our work represents a key step towards the standardization of automated fetal brain biometry reporting, addressing a critical gap in current clinical practice by reducing reliance on manual approaches and ensuring accurate and reproducible measurements to enhance the full clinical report. Furthermore, the end-to-end pipeline can overcome the practical challenges to widespread clinical integration, which would enable more centers to undertake good-quality fetal MRI, as well as increasing accessibility to a broader population.

### Limitations and future work

While we have demonstrated the initial feasibility of automated biometry for fetal brain MRI, it represents only a part of the full clinical report and additional extensive further work is needed before it is ready for routine integration into clinical practice. This includes incorporating abnormal cases with structural anomalies and cases with varying imaging quality to ensure robustness, as well as validating on external datasets with diverse patient populations to improve generalizability. Furthermore, expanding the scope to include craniofacial biometry [26] as well as volumetric information and 3-D surface-based biometry measurements based on segmentations [31] could enhance its clinical utility, enabling more precise quantification of brain development as well as improved detection and characterization of abnormalities. Additionally, an automated quality control system would also need to be implemented to detect issues, failure modes, and model performance drift. We would also need to correlate automated measurements with neurodevelopmental outcomes to establish their added value in clinical decision making and prognostication, and explore the influence of sex, parental characteristics, ethnicity differences, and other determinants which can impact normal fetal growth. Such variables are critical for assessing the robustness of the automated system and ensuring its applicability across diverse populations.

## Conclusion

This work introduces the first prototype for automated brain biometry and centile calculation for 3-D motion-corrected T2w fetal MRI within a clinical reporting pipeline. The quantitative and qualitative evaluation on 90 and 111 datasets from different acquisition protocols and wide GA range confirmed the general feasibility and utility of the pipeline as a decision support tool for clinical interpretation. We also generated normative growth charts for 19-40 weeks GA from 406 control datasets. Future work will focus on optimization for various fetal brain anomalies, extending the set of measurements, developing automated quality control, and external validation of the reporting tool.

## Data Availability

The fetal MRI data used in this work were acquired as part of the ethically approved research studies: "Placental Imaging Project" (REC 16/LO/1573), "Individualised Risk prediction of adverse neonatal outcome in pregnancies that deliver preterm using advanced MRI techniques and machine learning" (REC 21/SS/0082), "CARP" (REC 19/LO/0852), "MEERKAT" (REC 21/LO/0742), "MiBirth" (REC 23/LO/0685), "NANO" (REC 22/YH/0210), and "Quantification of fetal growth and development with MRI" (REC 07/H0707/105) in accordance with the ethical standards as laid down in the 1964 Declaration of Helsinki and its later amendments. These studies have research ethics committee (REC) approval by the Health Research Authority boards of the following: London (including -Fulham, -South East, -Riverside, Dulwich, -West London and GTAC, and -Brent) and South East Scotland. Contact address: Health Research Authority, 2 Redman Place, Stratford, London, E20 1JQ. The individual fetal MRI datasets used for this study are not publicly available due to ethics regulations. For more information please contact Jana Hutter jana.hutter@kcl.ac.uk.

## Footnotes

1 auto-proc-SVRTK toolbox: https://github.com/SVRTK/auto-proc-svrtk

2 ITK-SNAP tool: http://www.itksnap.org/

3 MIRTK toolbox: https://github.com/BioMedIA/MIRTK

4 SVRTK docker: https://hub.docker.com/r/fetalsvrtk/svrtk

5 auto-SVRTK toolbox: https://github.com/SVRTK/auto-proc-svrtk

6 3-D Slicer platform: https://www.slicer.org/

7 MITK Workbench platform: https://docs.mitk.org/2024.06/MITKWorkbenchManualPage.html
